# Anterior Interosseous Nerve Neuropraxia Secondary to Shoulder Arthroscopy and Open Subpectoral Long Head Biceps Tenodesis

**DOI:** 10.1155/2017/7252953

**Published:** 2017-04-16

**Authors:** Jeremiah T. Steed, Kathlyn Drexler, Adam N. Wooldridge, Matthew Ferguson

**Affiliations:** Texas Tech University Health Sciences Center, Department of Orthopaedic Surgery and Rehabilitation, 3601 4th St. MS 9436, Lubbock, TX 79430, USA

## Abstract

Arthroscopic rotator cuff tendon repair is a common elective procedure performed by trained orthopaedic surgeons with a relatively low complication rate. Specifically, isolated neuropraxia of the anterior interosseous nerve (AIN) is a very rare complication of shoulder arthroscopy. An analysis of peer-reviewed published literature revealed only three articles reporting a total of seven cases that describe this specific complication following standard shoulder arthroscopic procedures. This article reports on three patients diagnosed with AIN neuropraxia following routine shoulder arthroscopy done by a single surgeon within a three-year period. All three patients also underwent open biceps tenodesis immediately following completion of the arthroscopic procedures. The exact causal mechanism of AIN neuropraxia following shoulder arthroscopy with biceps tenodesis is not known. This case report reviews possible mechanisms with emphasis on specific factors that make a traction injury the most likely etiology in these cases. We critically analyze our operating room setup and patient positioning practices in light of the existing biomechanical and cadaveric research to propose changes to our standard practices that may help to reduce the incidence of this specific postoperative complication in patients undergoing elective shoulder arthroscopy with biceps tenodesis.

## 1. Background

Anterior interosseous nerve (AIN) neuropraxia, also known as anterior interosseous syndrome, presents clinically as the inability to flex the distal phalanges of the thumb and index finger as well as possible weakness in pronation, all resulting from the paralysis of flexor pollicis longus (FPL), flexor digitorum profundus (FDP) to the index finger, and pronator quadratus (PQ).

**Figure 1:**
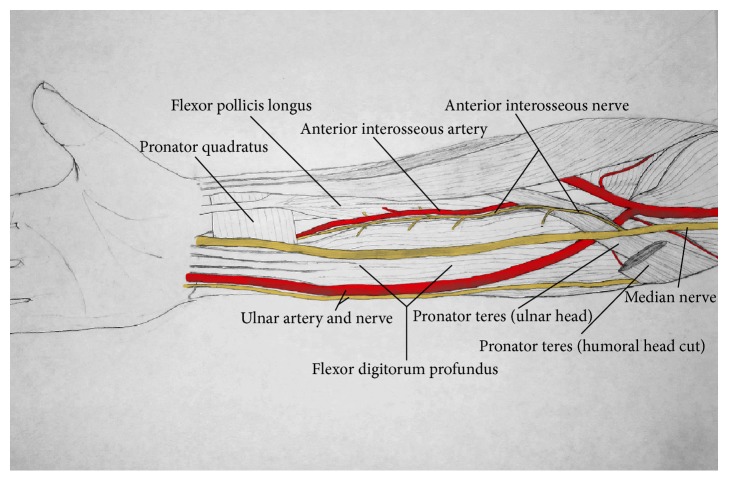
Rendition of forearm anatomy ~ drawn by Jeremiah Steed.

The median nerve is formed by branches of the medial and lateral cords of the brachial plexus. It courses down the arm adjacent to the brachialis muscle, crosses the antecubital fossa under the lacertus fibrosus, and then travels between the superficial and deep heads of the pronator teres muscle. At this point, prior to passing deep to the fibrous arch of the flexor digitorum superficialis, the median nerve bifurcates into the median nerve proper and the AIN (Figure 1).

Historically, AIN neuropraxia has been shown to arise spontaneously, as a result of trauma, or as a component of brachial neuritis [1]. In 1948, Parsonage and Aldren Turner were first to report on shoulder-girdle syndrome, now better known as brachial neuritis [2]. Isolated AIN neuropraxia has since been shown to be one possible clinical presentation of brachial neuritis [3–5]. In 1952, Kiloh and Nevin described an isolated AIN neuritis that occurred spontaneously and independent of any known precipitating condition [6].

AIN neuropraxia as a possible complication of shoulder arthroscopy is a scarce topic in the literature. To our knowledge, only three articles [7–9] have been published describing this specific condition following shoulder arthroscopy. This article reports three instances of AIN neuropraxia following shoulder arthroscopy with open tenodesis and explores possible mechanisms by which this condition could result secondary to this procedure.

## 2. Case 1

A thirty-seven-year-old male presented with persistent right should pain after a car accident 2 months earlier. The patient was a nonsmoker with a BMI of 32, a history of dyslipidemia and hypertension, and no prior shoulder problems. Initial physical exam revealed good strength in the flexor pollicis longus, flexor digitorum profundus, and intrinsic muscles of the hand, but the patient had pain upon resisted external rotation and abduction of the shoulder. He had pain with palpation over the bicep tendon and deltoid insertion. MRI revealed a partial thickness supraspinatus tear along with fluid around the biceps tendon. The patient was diagnosed with a partial rotator cuff tear, bicep tendonitis, and a deltoid strain. Surgery was scheduled after he had failed conservative treatment which included a corticosteroid injection and physical therapy for two months without significant improvement. On the day of surgery, the patient was given a preoperative, ultrasound-guided, interscalene nerve block with 25 mL of 0.5% ropivacaine administered with a 21 gauge, 90 mm, echogenic short bevel needle. Next, the patient underwent induction of general endotracheal anesthesia. The patient was placed in the lateral decubitus position with the head in a neutral position, and a boom was used to apply 15 pounds of traction throughout the surgery. Glenohumeral debridement and a subacromial bursectomy were performed arthroscopically, and arthroscopic inspection revealed extensive synovitis along the long head of the biceps tendon. Following the arthroscopic procedure, the weighted traction was released and an open subpectoral long head biceps tenodesis was performed. The total case time was one hour and thirty-eight minutes.

One week postoperatively, the patient presented with difficulty flexing the DIP joint of the index finger and the IP joint of his thumb. At three weeks postoperatively, the patient was found to have profound weakness of FPL and FDP and he was diagnosed with AIN palsy of the right hand. An electromyography and nerve conduction study was done seven weeks postoperatively and revealed no major abnormalities in the median nerve proper, suggesting that the insult was primarily isolated to the AIN nerve. At eight weeks postoperatively, the patient's AIN palsy was clinically improving and weakness had decreased.

## 3. Case 2

A thirty-one-year-old male presented with a six-month history of right shoulder pain with insidious onset after repetitive work-related shoulder exercises and movements. The patient was a nonsmoker with a BMI of 28. He had a history of Hashimoto thyroiditis and a right shoulder rotator cuff tear repaired arthroscopically in 1997. Upon physical examination, the patient had full strength of FPL, FDP, and the intrinsic muscles of the hand and pain with resisted external rotation as well as tenderness with palpation over the bicep tendon. MRI revealed significant fluid collection in the bicipital groove surrounding the long head of the biceps tendon and a subchondral lesion in the greater tuberosity of the humerus suggestive of a cyst. The patient was diagnosed with biceps tenosynovitis and a superior labrum tear with shoulder impingement. Surgery was scheduled after the patient failed conservative treatment options including physical therapy and corticosteroid injections having no significant improvement in his symptoms. On the day of surgery, the patient was given a preoperative, ultrasound-guided, interscalene nerve block with 25 mL of 0.5% ropivacaine administered with a 21 gauge, 90 mm, echogenic short bevel needle. Next, the patient underwent induction of general endotracheal anesthesia. The patient was placed in the lateral decubitus position with the head in a neutral position, and a boom was used to apply 15 pounds of traction throughout the arthroscopic procedure. Glenohumeral debridement and a subacromial bursectomy were performed arthroscopically. The weighted traction was then released and an open subpectoral long head biceps tenodesis was performed. The total case time was exactly 2 hours.

The patient returned to clinic twelve days postoperatively with symptoms consistent with AIN palsy of the right hand along with median nerve involvement at the level of the wrist. He was unable to flex the MCP or IP joints of the thumb or index finger, but flexion of the 3rd, 4th, and 5th digits remained intact. The patient also had a positive Tinel's sign over the carpal tunnel as well as a positive Phalen's test. At ten weeks postoperatively, the patient's AIN palsy had somewhat improved but he had residual weakness in flexion of the index finger DIP and thumb IP joints. An electromyography and nerve conduction study was done six weeks postoperatively and revealed an injury proximal to the pronator teres.

At thirteen months postoperatively, when the patient's symptoms had failed to improve, a surgical exploration of the median nerve and anterior interosseous nerve was performed along with neurolysis of these nerves at the antecubital fossa and a release of the median nerve at the carpal tunnel. Intraoperatively, dense fascial bands in the forearm were observed to be pinching and these were released. Within 2 months following the release of the median nerve at multiple levels, the patient's symptoms began to improve.

## 4. Case 3

A fifty-year-old male presented with a one-month history of left shoulder pain unresponsive to conservative management. The patient was a nonsmoker with a BMI of 27, a history of dyslipidemia, and no prior history of shoulder problems. Upon physical exam the patient had decreased ROM of the left arm as well as a positive Jobe sign, Hawkins sign, and Speed test. An MRI was consistent with a large, full thickness rotator cuff tear with fatty infiltration of the supraspinatus and infraspinatus. The patient was diagnosed with a full thickness rotator cuff tear and opted for surgical management. On the day of surgery, the patient was given a preoperative, ultrasound-guided, interscalene nerve block with 25 mL of 0.5% ropivacaine administered with a 21 gauge, 90 mm, echogenic short bevel needle. Next the patient underwent induction of general endotracheal anesthesia. The patient was placed in the lateral decubitus position with the head in a neutral position, and a boom was used to apply 15 pounds of traction throughout the arthroscopic procedure. Glenohumeral debridement and subacromial bursectomy were performed arthroscopically, followed by repair of the supraspinatus and infraspinatus rotator cuff tendons. Inspection of the biceps tendons revealed extensive synovitis. Traction was released and an open subpectoral long head biceps tenodesis was performed. The total case time was three hours and twenty-eight minutes.

The patient presented seven weeks postoperatively with the inability to flex the left thumb interphalangeal joint or the index DIP joint; he was diagnosed with AIN palsy. The patient also had mildly decreased sensation to light touch along the median nerve distribution in the hand and mildly decreased sensation to light touch and subjective paresthesias along the ulnar nerve distribution. However, according the patient, the findings along the ulnar nerve distribution existed prior to the surgery. He was observed clinically and at twenty-two weeks postoperatively began to show improvement by demonstrating motor recovery of the AIN with flexion of the thumb IP joint; at this time he still lacked index finger DIP flexion. At twenty-eight weeks postoperatively, an electromyography and nerve conduction study was attempted, but the patient did not tolerate and therefore did not complete these studies. However, the limited results available were suggestive of a severe proximal median neuropathy, as well as findings suggestive of a possible C8 radiculopathy, likely related to the history of prior ulnar nerve dysfunction.

## 5. Discussion

The etiology of the AIN palsies after arthroscopic shoulder surgery in the patients in this report is unclear. The surgical incisions in such procedures are not in close proximity to the normal AIN bifurcation from the median nerve. Although evidence is limited, previous publications have suggested a traction injury during surgery as the most plausible etiology, with other possible mechanisms being compression from the traction sleeve or a complication of the interscalene block [7–9]. In addition to the above mechanisms, the patients in this report share in common an open biceps tenodesis following the arthroscopic procedure.

Since the initial description of brachial neuritis by Parsonage and Aldren Turner in 1948 [2], it has since been reported that brachial neuritis can present clinically as isolated AIN syndrome in the absence of other more global brachial plexus involvements [3]. Furthermore, there have been many reports of interscalene blocks associated with brachial neuritis [10–13]. Therefore, it is possible that the interscalene blocks in the three patients presented above could have precipitated a brachial neuritis that presented as AIN neuropraxia. However, in our patients, none of the nerve conduction studies revealed diffuse proximal brachial plexus abnormalities, which would be expected if brachial neuritis were the underlying cause [3]. This observation makes an anesthesia-induced brachial neuritis a less likely etiology in these patients.

Another possible etiology is palsy of the AIN secondary to ischemia. None of the patients in this report experienced any prolonged episodes of intraoperative hypotension or bradycardia. However, traction used for shoulder arthroscopy has been shown to decrease tissue perfusion in the upper extremity. One study, which used pulse oximetry to measure tissue perfusion, revealed that traction during shoulder arthroscopy can attenuate tissue perfusion to the distal extremity [14]. This could lead to transient local ischemia of the AIN. AIN palsy precipitated by transient ischemia has been reported. In the literature, one article described three cases of supracondylar fractures in children which were followed by palsy of the AIN and proposed transient ischemia of the nerve as the probable cause [15]. All three of these children had weak/absent radial pulses upon presentation and normal neurological exams after manipulation of the fracture, followed by a delayed onset of AIN palsy. Though the 15 lbs of weight and the short duration of traction used during shoulder arthroscopy are unlikely to cause a significant ischemic episode, damage by a similar mechanism has been shown to occur. While this alone may not have independently precipitated the AIN palsies seen in the patients in this report, transient ischemia could play a role by adding insult to the nerve.

**Figure 2:**
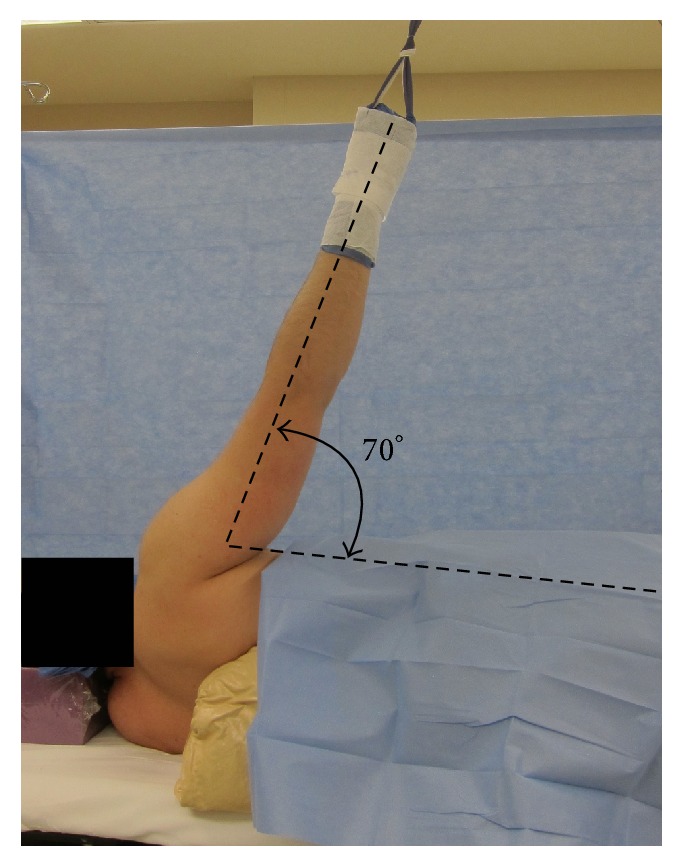
Recreation of the setup used in the patients in this report demonstrating 0° of flexion and 70° of abduction.

A traction injury due to patient positioning during shoulder arthroscopy is another possible etiology of AIN neuropraxia secondary to shoulder arthroscopy. One cadaveric study done by Klein Alan et al. showed that longitudinal traction of the shoulder with the patient in the lateral decubitus position placed strain on many of the nerves in the upper extremity, including the median nerve [16]. The study also showed that the true strain on the nerves was inversely proportional to the cross-sectional area of the nerve being measured, meaning that smaller nerves experienced a greater change in length in response to traction. Because the AIN branch is smaller than the median nerve proper, it could be at greater risk for damage. A study done by Pitman Mark et al. showed that 15 lbs of longitudinal traction with the patient in the lateral decubitus position could cause abnormal somatosensory evoked potentials in nerves of the upper extremity, including the median nerve [17]. A cadaveric study done by Collins David and Edward revealed that tension placed on the median nerve could actually put the AIN at greater risk for damage then the median nerve because of its limited mobility and small size compared to the median nerve [18]. A traction injury to both the median nerve and the AIN branching from it could explain the involvement of both of these nerves observed in our second and third patients. The cadaveric study done by Klein Alan et al. measured the strain on the brachial plexus during traction in 17 different positions and compared this to the visibility offered by each position during shoulder arthroscopy [16]. They showed that although the position of 30° of forward flexion and 70° of abduction offered excellent visibility of the entire shoulder joint, this was also the position that placed the overall greatest strain on the brachial plexus. During the recreation of the setup used for the patients in this report, we found that the position used was approximately 0° of forward flexion and 70° of abduction (Figure 2). The study done by Klein Alan et al. would suggest that this position is associated with relatively high strain on the median nerve compared to other recommended positions.

Also of interest is that all three of our patients who developed a postoperative AIN neuropraxia had an open long head biceps tenodesis in addition to shoulder arthroscopy. It is possible that the extended operation time and additional manipulation required for the bicep tenodesis could have played a role in the observed AIN palsies. However, our literature review did not reveal any cases or investigations regarding this as a mechanism of AIN injury.

**Figure 3:**
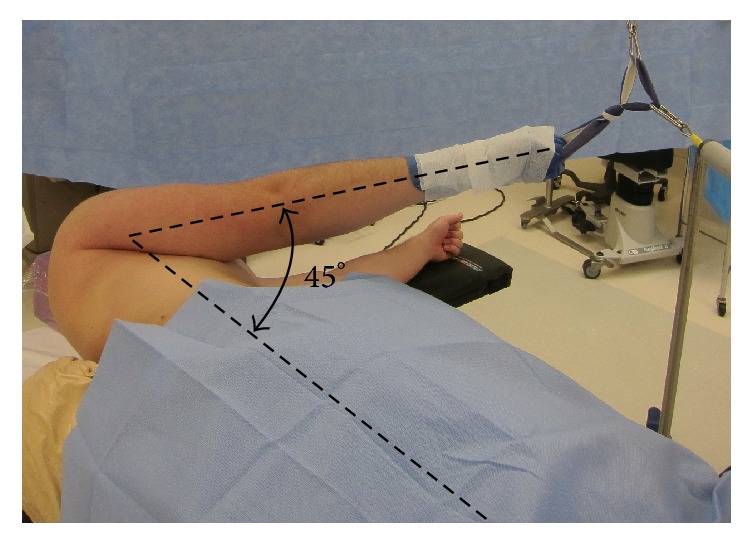
Recreation demonstrating 45° of flexion and 0° of abduction.

**Figure 4:**
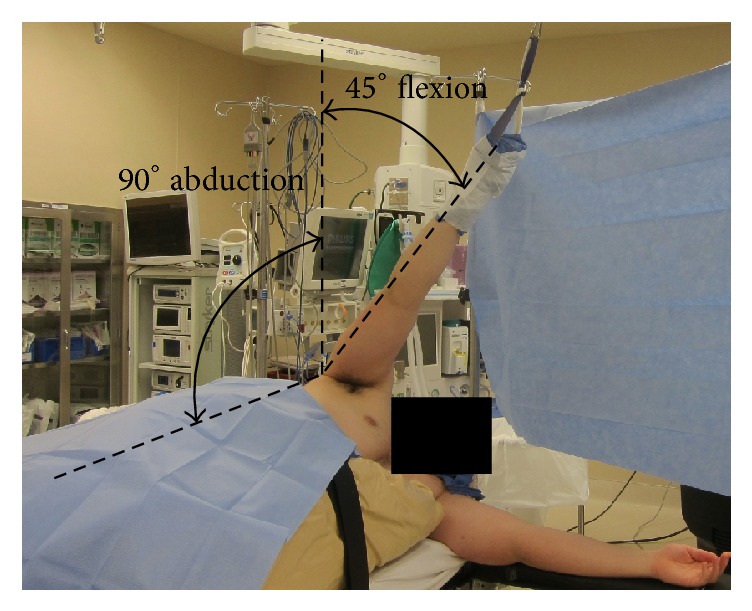
Recreation demonstrating 45° of flexion and 90° of abduction.

Due to our current positioning practices during standard shoulder arthroscopy, we have concluded that a traction injury is the most likely etiology of the AIN palsies observed in the patients in this report. We have reviewed the literature in an attempt to identify practice changes that could lower the risk of postoperative AIN neuropraxia. Careful patient positioning during shoulder arthroscopy is an important consideration for decreasing the incidence of traction injuries postoperatively. The ideal position would be one which minimizes strain on neurovascular structures while allowing for adequate visualization and accessibility of the shoulder joint. Klein Alan et al. found that no one position offered good visibility of the entire shoulder joint while at the same time minimizing strain on the brachial plexus [16]. However, they did find that by switching between two positions, the entire shoulder joint could be adequately visualized while maintaining minimal strain on neurovascular structures. The position of 45° of forward flexion and 0° of abduction in combination with the position of 45° of forward flexion and 90° of abduction allows for good visibility and minimizes strain on the brachial plexus (Figures 3 and 4).

In addition to the direction of traction, the amount of weight used during traction is another important consideration for decreasing the incidence of traction injuries. As expected, the study by Klein Alan et al. showed that increasing traction weight increased the strain on the brachial plexus in all positions tested [16]. The standard practice of the primary surgeon during the time period in which these cases occurred was to place 10 lbs of traction and increase to 15 lbs intraoperatively if needed for visualization and access to joint structures. In the patients cited in this report, 15 lbs of traction was used during surgery. Further research is needed to define the relationship between traction weight and increased risk of neuropraxia.

AIN neuropraxia is a rare complication of shoulder arthroscopy, but one with which surgeons should be familiar. It is important that surgeons are aware of potential injuries that may occur due to traction and positioning. Utilization of different traction positions that minimize strain placed on the brachial plexus could help reduce the incidence of postoperative neuropraxia in the future.
